# The collaborative learning development exercise (CLeD-EX): an educational instrument to promote key collaborative learning behaviours in medical students

**DOI:** 10.1186/s12909-020-1977-0

**Published:** 2020-03-02

**Authors:** Maha Pervaz Iqbal, Gary M. Velan, Anthony J. O’Sullivan, Chinthaka Balasooriya

**Affiliations:** 1 School of Public Health and Community Medicine, UNSW Medicine, UNSW Sydney, Sydney, Australia; 2 School of Medical Sciences, UNSW Medicine, UNSW Sydney, Sydney, Australia; 3 St. George and Sutherland Clinical School UNSW Sydney, Sydney, Australia

**Keywords:** CLeD-EX, Collaborative learning, Medical students, Educational instrument, Student development

## Abstract

**Background:**

Modern clinical practice increasingly relies on collaborative and team-based approaches to care. Regulatory bodies in medical education emphasise the need to develop collaboration and teamwork competencies and highlight the need to do so from an early stage of medical training. In undergraduate medical education, the focus is usually on collaborative learning, associated with feedback and reflection on this learning This article describes a novel educational instrument, the Collaborative Learning Development Exercise (CLeD-EX), which aims to foster the development of key collaborative learning competencies in medical students. In this article we report on the effectiveness, feasibility and educational impact of the CLeD-EX.

**Methods:**

In this study, the “educational design research” framework was used to develop, implement and evaluate the CLeD-EX. This involved adopting a systematic approach towards designing a creative and innovative instrument which would help solve a real-world challenge in developing collaborative learning skills. The systematic approach involved a qualitative exploration of key collaborative learning behaviours which are influential in effective collaborative learning contexts. The identified competencies were employed in the design of the CLeD-EX. The design of the CLeD-EX included features to facilitate structured feedback by tutors to students, complemented by self-evaluation and reflection. The CLeD-EX was field-tested with volunteer junior medical students, using a controlled pre-test post-test design. Analysis of the completed CLeD-EX forms, self-perception surveys (i.e. pre-test and post-test surveys) and analyses of reflective reports were used to explore the educational impact of CLeD-EX, as well as its utility and practicality.

**Results:**

After using the CLeD-EX, students showed a significant improvement in critical thinking and group process as measured by a previously validated instrument. Both students and tutors recognised CLeD-EX as an effective instrument, especially as a structured basis for giving and receiving feedback and for completing the feedback loop. CLeD-EX was also found to be feasible, practical and focused, while promoting learning and effective interactions in small group learning.

**Conclusion:**

The findings of this study support the introduction of an effective and feasible educational instrument such as the CLeD-EX, to facilitate the development of students’ skills in collaborative learning.

## Background

Collaborative learning and teamwork are core competencies that impact on the quality of health care [1]. Accreditation bodies for Medicine programs therefore, have developed frameworks to ensure that collaboration and teamwork skills are developed to standards required for effective clinical practice and patient care [2–4]. Globally, medical schools are urged to place an emphasis on developing these skills from an early stage [5]. At the start of a medicine program, medical students often work and learn together in small group learning contexts, elevating the importance of collaborative learning. Collaborative learning is broadly defined as “students working in groups of two or more, mutually searching for (*knowledge*) understanding, solutions, or meanings, or completing a task or creating a product. There is wide variability in collaborative learning activities, but most centre on the students’ exploration or application of the course material, not simply the teacher’s presentation or explication of it (*content*)” [6]. Therefore, during collaborative learning students have the opportunity to develop skills in managing their learning (i.e. self-directed learning) while interacting with peers to discuss and debate concepts which will promote higher order cognitive reasoning.

Despite the use of a variety of approaches such as small group learning activities (e.g. Problem based learning, team-based learning, etc) or group assessments (e.g. group projects), promoting effective collaborative learning in medical students continues to be a challenge. Simply putting students in small groups and expecting them to collaborate and learn together does not automatically lead to effective collaborative learning. Research has highlighted several challenges that students experience in collaborative learning such as unequal individual participation in group work [7], lack of effective communication [8] free-riding and social loafing on collaborative tasks [9] and dealing with difficult and dominant group members [10]. These challenges can be due to inexperience in working in groups, the lack of clarity around the purpose of the group work or due to the lack of collaborative learning skills among medical students.

It has been proposed that medical students would benefit from receiving structured guidance for learning and interacting within small group, collaborative contexts. Importantly, medical students also identify the need for learning interactional skills relevant for collaboration and teamwork [11]. Moreover, key recommendations from the literature include regular evaluation and the use of formative rather than summative assessments of collaborative group work, or using self- and peer evaluations to promote the students’ skills for effective collaborative learning [12]. Previous research of using peer evaluation in formative assessment of student behaviours in small group learning activities has reported it as a useful process for providing feedback to students [13]. A recent systematic review has shown that peer feedback in collaborative learning environments can aid in the development of students’ professional behaviours [14]. Moreover, self-evaluation can be valuable in group work assessment especially when used for the purpose of formative assessment [15]. Self-evaluation also allows students the opportunity to identify their learning needs while taking increased responsibility for their learning, i.e. developing their skills in self-directed learning [16]. Although tutor evaluation is considered superior to self and peer evaluation in small group work [17]; there are concerns related to feasibility when tutors are expected to provide individual students with meaningful and timely feedback on their performance [18]. Therefore, providing a structured framework for evaluation and feedback by tutors could help overcome the challenges tutors face in providing useful and timely evaluation and feedback to students during collaborative learning activities.

Research has identified several instruments which have been used to assess and promote collaborative learning in small group environment [19–31]. There are several limitations identified in these instruments. Firstly, the content focus of these instruments was variable: some instruments focused on interactional skills, while others focused more on learning skills. Moreover, these instruments were limited by the absence of a clear format for tutors to provide feedback and the absence of a structure to guide student reflection on the feedback received. Few of these identified instruments [20, 21, 26, 28] reported on the practicality and feasibility of use in busy educational contexts.

The literature reviewed above highlighted the need to develop an instrument that would be feasible and practical in busy educational contexts. The Collaborative Learning Development-Exercise (CLeD-EX) was developed to address this need. The design of the CLeD-EX included a structured format for evaluation, feedback and reflection to promote key behaviours for effective collaborative learning in medical students. In this manuscript we report on the effectiveness, feasibility and educational impact of the CLeD-EX.

## Methods

### Research which led to the development of CLeD-EX

The “educational design research” framework [32, 33] was used to develop, implement and evaluate the CLeD-EX. This involved adopting a systematic approach towards designing a creative and innovative instrument which would help solve real-world challenges in developing collaborative learning skills in junior medical students. The details of this work have been reported by the authors in prior publications [34, 35]. The systematic approach involved a qualitative exploration of key collaborative learning behaviours which are influential in promoting effective collaborative learning [34]. In the next step, a modified Delphi study was conducted and the top six, key student behaviour which were influential in promoting effective collaborative learning were identified [35]. All the experts involved in the two-round Delphi (round 1 *n* = 54; round 2 *n* = 23) had an academic role in universities across Australia and New Zealand and were involved in small-group teaching that included design and/or facilitation of collaborative learning. These six behaviours were included in the CLeD-EX Instrument (Table 1).

**Table 1:** Key collaborative learning behaviours for inclusion in the design of the CLeD-EX instrument [35]

Key collaborative learning behaviours for inclusion in the design of the CLeD-EX instrument	
(The student) Is well prepared for the learning session;	
(The student) Is involved in discussion and debate on different ideas;	
(The student) Appears willing to work and is engaged in the learning;	
(The student) Listens to others’ points of view;	
(The student) Shares information with group members and voices own opinions;	
(The student) Reflects on feedback and responds appropriately to it	

### Design of the CLeD-EX instrument

The CLeD-EX consisted of an evaluation and feedback component. A four-point frequency scale was selected for the evaluations, with response options: never; rarely; often; and always. A fifth option of unable to assess was also included along with a space to provide a comment. The administration of the CLeD-EX was structured as follows:

Stage: 1 Student self-evaluation: Each student rates themselves in relation to the behaviours on the CLeD-EX in the context of small group learning.

Stage: 2 Tutor evaluation and feedback: In this part, tutors, who are all academics in health professions education, evaluate the behaviours of each student during small group sessions. Tutors, while facilitating small group sessions, observe the frequency of the behaviours that students exhibit during the learning activities. Space is allocated for tutors to provide feedback on two aspects: “Positive aspects of collaboration”; and “Areas that can be improved.” The written feedback is further supplemented by a dialogue between the tutor and the student. The purpose of this oral feedback is to elaborate on the written feedback and discuss an action plan for improvement.

Stage: 3 Student reflections: In this section of the form, each student reflects on the entire activity and writes a reflective account on the following two aspects: “Issues which were raised in this exercise that need to be addressed (to improve my collaborative learning behaviours);” and “Action plan for improvement.”

The above design illustrates how the key tenets for effective feedback which includes self-evaluation; feedback; and feedforward [36] were incorporated in the CLeD-EX.

Two global items were included at the end of the self and tutor evaluations. The first global item focused on key collaborative behaviours and their impact on the quality of learning. The second item obtained the student’s and tutor’s perceptions of the overall collaborative skill of the learner.

Information was provided to both students and tutors regarding the purpose of the CLeD-EX, including provision of feedback to assist development and encouragement of reflection. Tutors were asked to indicate the amount of time it took to provide feedback (this was an important consideration related to the feasibility of the CLeD-EX). Following completion of the student reflection section of the form, students were asked to respond to two items relating to the usefulness of the CLeD-EX and the feedback received through the instrument.

The final design of CLeD-EX was reviewed for clarity, by the members of the project team and three tutors of small group learning at UNSW Medicine. The tutors were introduced to the CLeD-EX through a brief presentation in the course orientation session. The purpose of this presentation was to introduce the tutors to the CLeD-EX and to address any queries about the instrument or the field-test design. The CLeD-EX was implemented via a fillable (smart) PDF form, which was emailed to students, completed online and saved in electronic form, then returned by email. In addition, students could print the form and complete it in pen and paper format if they preferred.

### Field-test of CLeD-EX

The context for the CLeD-EX field-test was the scenario group learning sessions which are key components of Phase-1 (years 1 and 2) of the UNSW Medicine program. Scenario group learning is a variant of problem-based learning, designed and utilised at UNSW Medicine [37]. Students, in groups of 14–15 facilitated by a tutor, spend two hours twice-weekly working on structured collaborative learning activities, presentations and tasks around relevant clinical scenarios.

**Fig. 1:**
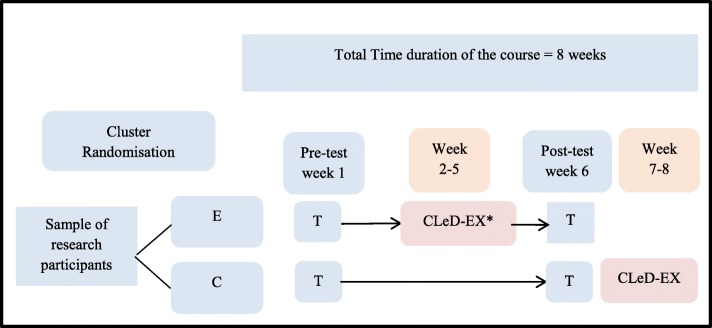
Format of Field-test for CLeD-EX

The field test of the CLeD-EX involved an experimental format with a pre and post-test (Fig. 1). All students in two different courses in Phase 1 were invited to voluntarily participate in the CLeD-EX field-test. Students who agreed to participate were randomly allocated into control and experimental groups. At this stage, cluster randomisation was performed using existing structures of the Medicine program (whereby students are randomly assigned into four colleges, i.e. A, B, C and D, for administrative purposes). Small group, collaborative learning activities are scheduled at separate times for each college; the colleges were considered as separate clusters for this study. Volunteer students were randomised into control and intervention groups based on their colleges (i.e. clusters). The rationale for this cluster randomisation trial design was to avoid cross-contamination between the control and intervention groups within small group learning activities [38]. However, for equity, the control group also received a copy of the CLeD-EX after completion of the study.

E- Experimental group, C-Control group, T- Self-perception test, CLeD-EX Instrument.

*Students complete the CLeD-EX instrument once during the 8 weeks duration of the course. Students in the intervention group had the opportunity to complete CLeD-EX during weeks 2, 3, 4, and 5. While students in the control group received CLeD-EX after completing the post-test; students in the control group completed CLeD-EX during weeks 7 and 8 of the course.

### Evaluation of CLeD-EX forms

All the CLeD-EX forms received after completion of the study were analysed. The rating on the forms were scored from 1 to 4 (1 = Never, 2 = Rarely, 3 = Often, 4 = Always). The response ‘unable to assess’ was handled as missing data. The analysis involved the following aspects. Firstly, the internal consistency (reliability) of CLeD-EX items was analysed using Cronbach’s alpha for the self and tutor evaluation scales. Secondly, comparison of self-evaluation with tutor evaluation was analysed through comparison of means and relative effect sizes. The practical significance of statistically significant differences between means were verified using effect sizes, calculated using Cohen’s D. Inter-rater agreement was calculated using Cohen’s Kappa. Thirdly, the acceptability and feasibility of CLeD-EX was measured based on the time taken for tutors to complete evaluation and feedback forms, as well as students’ responses to two items at the end of the form. Finally, a qualitative analysis was conducted of open-ended feedback and student reflections. The written feedback was analysed using a modified version of a framework originally developed to evaluate written feedback on a workplace-based rating form [39]. The taxonomy applied in the analysis of the written comments on the CLeD-EX involved the following categories: identifying the behaviour, identifying the performance gap and providing advice for developing a learning plan. Each component in the CLeD-EX form was scored dichotomously, absent (score = 0) or present (score = 1). The first author (MPI) analysed all the written comments based on this algorithm, while the research team (GV, AOS and CB) analysed a random sample of the written comments. Any discrepancies were discussed and resolved. The depth and breadth of student discussion in the student reflections was analysed to see if the CLeD-EX triggered a change in their thinking and/or understanding and encouraged them to plan an action to improve or to act differently.

### Pre-test and post-test evaluation

The pre and post-test was conducted using a previously validated questionnaire designed by researchers at McMaster University [40] to measure improvement in self-directed learning, critical thinking and group process. All students participating in the CLeD-EX field-test completed the pre and post-test questionnaire. The 29 items of the instrument are classified into three subscales: self-directed learning (SDL); critical thinking (CT); and group processing (GP). The internal consistency reliability of these subscales was confirmed in this study with Cronbach alphas for each subscale as follows: SDL = 0.853; CT = 0.7995; and GP = 0.855. The response option for the items was on a six-point, ordinal Likert-type scale 0 = never; 1 = almost never; 2 = sometimes; 3 = often; 4 = almost always; and 5 = always. The score for each subscale was the total score of all items in that scale (a higher score represented a better outcome). Students completed the pre and post-tests online via the Key Surveys (UNSW: WorldAPP-Key Surveys -Online survey tool).

An independent sample t-test was conducted to explore the differences between the intervention and control groups in the pre-test and post-test. A paired samples t-test was conducted to evaluate change in the intervention group following the use of the CLeD-EX. A Preliminary analysis was conducted to ensure that there was no violation of the assumptions of normality. All quantitative data analysis was conducted using the statistical software package, IBM SPSS version 24.

### Ethics

Ethics approval for this study was obtained from Human Research Ethics Advisory Panel (HREA) UNSW Sydney (Reference number: 2014-7-39).

## Results

All Phase 1 students, across two courses (*n* = 840) were invited to participate in the CLeD-EX field-test. A total of 107 students volunteered to participate and completed the online pre and post-test (response rate: 12.7%). Within that cohort, 52 students were in the intervention group (Colleges A and D) while 55 were in the control group (Colleges B and C). Each scenario group included approximately 2–4 students who had volunteered to participate in the CLeD-EX field-test. All students were encouraged to submit the completed CLeD-EX forms; 93 completed CLeD-EX forms were received and analysed.

### Internal consistency reliability of the CLeD-EX

Cronbach’s alpha for the tutor-evaluation scale was 0.766 and for the self-evaluation scale the Cronbach alpha was 0.451. In both the self-evaluation and tutor evaluation scale, there was a small increase in Cronbach alpha (self-evaluation 0.474; tutor evaluation 0.770) if the behaviour ‘I listen to others’ point of view’ was removed from the scale (Tables 2 and 3). However, it was decided to retain the behaviour in both scales because it had minimal impact on the scale reliability.

**Table 2:** Item-total statistics for the CLeD-EX behaviours for Self-evaluation Scale

Behaviour	Scale Variance if Item Deleted	Item-Total Correlation	Cronbach’s Alpha if Item Deleted
I am well prepared for the learning sessions	2.247	.208	.414
I am willing to work and I am engaged in learning activities	2.118	.288	.369
I am involved in discussion and debate on different ideas	2.142	.173	.439
I listen to others’ point of view	2.453	.086	.474
I share information with group members’ and voice my opinions	1.966	.339	.333
I reflect on the feedback I receive and respond appropriately	2.133	.244	.393

**Table 3:** Item-total statistics for the CLeD-EX behaviours for Tutor evaluation Scale

Behaviour	Scale Variance if Item Deleted	Item-Total Correlation	Cronbach’s Alpha if Item Deleted
This student is well prepared for the learning sessions	3.920	.474	.741
This student is willing to work and is engaged in learning activities	4.180	.415	.754
This student is involved in discussion and debate on different ideas	3.206	.611	.704
This student listens to others’ point of view	4.328	.339	.770
This student shares information with group members’ and voices his/her opinions	3.024	.741	.659
This student reflects on the feedback they receive and respond appropriately	3.981	.490	.738

### Comparison of self-evaluation with tutor evaluation

Item by item paired t-tests (Table 2) were undertaken to compare students’ self-evaluation with tutor evaluation. For all items, except the behaviour “…listens to other’s point of view”, students scored themselves significantly lower than their tutor. Tutors’ evaluations of students’ collaborative behaviours were significantly more positive than students’ self-evaluations for five out of six key behaviours (Table 4). On the CLeD-EX form there was a statistically significant difference between mean summed scores of self-evaluation (Mean = 19.4891; SD = 1.68049) and mean summed scores tutor evaluation (Mean = 21.0746; SD = 2.27845); t(92) = 1.335; *p* value ≤0.05, CI = − 2.20546, − 0.96554. Therefore, there is a significant difference in self and tutor evaluation. Furthermore, Cohen’s effect size value was high (d = 0.79), suggesting that difference has practical significance.

**Table 4:** Comparison of means between self and tutor evaluation and their level of significance

Behaviour	Mean for self-evaluation (95% Confidence Interval)	Mean for tutor evaluation (95% Confidence Interval)	*P*-value (two tailed)
(Student) well prepared for the learning sessions	3.06 (2.96, 3.16)	3.52 (3.41, 3.63)	.000*
(Student) willing to work and I am engaged in learning activities	3.43 (3.33, 3.53)	3.68 (3.58, 3.78)	.002*
(Student) involved in discussion and debate on different ideas	3.03 (2.90, 3.15)	3.31 (3.16, 3.45)	.000*
(Student) listens to others’ point of view	3.74 (3.64, 3.84)	3.74 (3.65, 3.83)	.892
(Student) shares information with group members’ and voice my opinions	3.15 (3.03, 3.27)	3.38 (3.24, 3.51)	.000*
(Student) reflects on the feedback I receive and respond appropriately	3.10 (2.99, 3.21)	3.41 (3.29, 3.53)	.024*

Inter-rater agreement: Cohen’s κ was computed to analyse the correlations between the summed scale scores of student and tutor evaluation. There was low agreement between student and tutor evaluation, κ = −.021, *p* = .54. Kendall’s Tau-b was conducted to measure correlation between scores of individual items which would demonstrate whether the self and tutor raters ranking are ranking the items similarly. The results are reported in Table 5.

**Table 5:** Correlations between self-evaluation and tutor evaluation by item

Behaviours (Self-evaluation versus tutor evaluation)	Kendall’s Tau-b correlation coefficient	*p* value
…well prepared for the learning sessions	0.141	0.166
…willing to work and engaged in learning activities	0.402	0.000*
…involved in discussion and debate on different ideas	0.474	0.000*
…listen to others’ point of view	0.127	0.226
…share information with group members’ and voice own opinions	0.347	0.000*
…reflect on the feedback received and respond appropriately	0.152	0.196

In the global, dichotomous item on the self-evaluation scale “overall my skills in collaborative learning are well-developed”, 87 students answered “yes” while six students answered “no.” On the tutor evaluation scale, the global item “Overall this student demonstrates skills which enhance the quality of collaborative learning” was answered positively by 88 out of 89 respondents.

### Effectiveness and educational impact of CLeD-EX

#### Pre-test and post-test evaluation: Impact of CLeD-EX on Students’ Self-Directed Learning (SDL), Critical thinking (CT), and Group Processing (GP)

Comparison of pre-test self-evaluations by students (Table 6) revealed no significant difference between the intervention and control groups.

**Table 6:** Independent t-test comparison between intervention and control group in the pre-test

Subscale	CLeD-EX Intervention group- mean score (*N* = 52) (SD)	Control Group- mean score (*N* = 55) (SD)	*P*- value
Pre-test self-directed learning	32.35 (6.19)	30.67 (5.97)	.158
Pre-test critical thinking	29.88 (5.26)	28.16 (5.86)	.114
Pre-test group process	35.90 (6.34)	34.44 (6.14)	.227

In the post-test comparison (Table 7), the intervention group scores were significantly higher than the control group for Self-Directed Learning, Critical Thinking and Group Process.

**Table 7:** Independent t-test comparison between intervention and control groups in the post-test

Subscale	CLeD-EX Intervention group- mean score(*N* = 52) (SD)	Control Group- mean score (*N* = 55) (SD)	*P*- value
Post-test self-directed learning	35.02 (5.46)	32.42 (6.53)	.027*
Post-test critical thinking	32.42 (5.00)	28.45 (5.36)	.000*
Post-test group process	38.02 (5.96)	35.15 (5.73)	.013*

As shown in Table 8, students in the intervention group showed a significant improvement in all three subscales between pre-test and post-test. Cohen’s d was calculated as 0.35 to 0.42 for each subscale in the intervention group, suggesting moderate practical significance. In the control group, there was a significant improvement in the self-directed learning subscale only (Cohen’s d = 0.30), which suggests low practical significance.

**Table 8:** Paired samples t-test between intervention groups and control groups pre and post-test instrument

Subscale	CLeD-EX Intervention group (*N* = 52)			Control group (*N* = 55)		
	Pre: mean (95% Confidence Interval)	Post: mean (95% Confidence Interval)	*P*- value	Pre: mean (95% Confidence Interval)	Post: mean (95% Confidence Interval)	*P*-value
Self-directed learning	32.35 [30.67, 34.03]	35.02 [33.25, 36.79]	.012*	30.69 [29.11, 32.27]	32.42 [30.98, 33.86]	.016*
Critical thinking	29.88 [28.45, 31.31]	32.15 [30.69, 33.61]	.028*	28.16 [26.61, 29.71]	28.45 [27.12, 29.77]	.686
Group process	35.90 [34.18, 37.62]	38.02 [36.46, 39.58]	.034*	34.44 [32.81, 36.06]	35.15 [33.57, 36.72]	.322

### Written feedback and student reflections on the CLeD-EX

#### Analysis of written feedback

Tutors included written feedback in the majority of the CLeD-EX forms (94.6%). Some forms included only comments about positive aspects related to collaboration (12.9%). When analysing the quality of feedback based on the modified framework [39] in the section on the form: ‘areas that can be improved’ the behaviour which needed to be improved was mentioned in 73 forms, while the gap was mentioned in 17 forms and advice on how to develop was included in 32 forms. This finding suggests that the CLeD-EX was successful in facilitating formative feedback by highlighting behaviours that need further development.

A brief review of the content of the written feedback suggested that the most common focus was on the students participating in discussions, for example, “*Try to engage more with your peers during small group activities by exploring and discussing issues with them,*” and “*I suggest you set yourself a goal of contributing one significant idea to the discussion per class.*” This was followed by the feedback on student preparation for the small group learning, for example: “*Improving preparation can be one approach you could take. This involves reading ahead, which can be useful in providing a sound basis for learning in each SG session.”* These findings are consistent with the students’ self-evaluation in which similar behaviours were scored the lowest, also on the tutor-evaluation scale. These findings suggest an important link between the students’ self-evaluation and tutor evaluation. The tutors’ written feedback also emphasises development of these behaviours in the student. Meanwhile, comments for improvement were mainly on aspects related to developing skills to learn more effectively in small group context.

#### Analysis of student reflection

In the 93 CLeD-EX forms received, reflections were recorded in 86 forms (92%). This indicates that the structure of the CLeD-EX appears to facilitate students to reflect on the collaborative learning experience. Students in their reflective accounts discussed issues which were being raised through this exercise and their action plans for improvement. In the written reflections relating to the latter, students clearly expressed their desire to improve their behaviour for collaborative learning. This change was observed through comments which indicated an intention to change by developing a new understanding which was gained through this activity.

“I think, in order for me to be able to be more involved in discussions, I need to deepen my knowledge of topics covered in BDGA, beyond lectures and SG (scenario group) activities. In order to do this, I will draw upon textbooks and the extra learning resources in Moodle. This will also allow me to increase my spontaneous engagement in group discussions, as my views will be more informed. I realise now that I should also be more involved in SG debates, and before I was probably too cautious of speaking too much, so I think finding the right balance is key. When an idea is generated by another member, I will be keen to pick it up and discuss it (with the) further” (C-255)

“Primarily, improving my collaborative learning behaviours requires a change in attitude. I need to be less afraid of being wrong when answering questions and remember that as my scenario group is simply a platform for exchanging ideas that I don't need to take personally any disagreements with my own opinions. In terms of practically aiding my capacity to contribute to class, I believe I should come to classes having pre-read the lesson material where possible and conducted background research into topics that are new or unfamiliar to me. This will allow me to pre-identify questions that I can pose to the class. Such preparation will help me to feel more confident in initiating discussion.” (C- 128)

In their reflective comments some students acknowledged the importance of collaborative learning and the usefulness of developing their collaborative learning behaviours. Through this process students have recognised its value for building their skills in collaboration and learning.

*“In terms of other aspects of collaborative learning, I will continue to be engaged in all teamwork activities and listen to the opinions of others. In particular, I think our collaborative learning in SGs (scenario groups) will be enhanced if quieter members are drawn out to voice their opinions, so I'll keep this is mind. In short, teamwork is a great way of enhancing the quality of our learning, and not only does it deepen our knowledge, but it develops our broader social interpersonal skills.” (C-255)*

### Analysis of utility and feasibility

Two items focussed on utility and feasibility of CLeD-EX were completed by each student after completing the last section (Part 3 Student Reflection) in the CLeD-EX form. For the first item, “I found the CLeD-EX helped improve my skills in collaborative learning”, 81.5% of students agreed. For the second item, “I was provided with useful feedback through the CLeD-EX process”, 92.3% of students responded positively.

The feasibility of the CLeD-EX was ascertained based on the time taken for tutors to give feedback to students. The mean time recorded for completing the evaluation and providing feedback was 6.25 min. The minimum time recorded by the tutor on the CLeD-EX to provide feedback was two minutes and the maximum time was 15 min. The majority of tutors recorded that the time taken to give feedback to student was five minutes.

## Discussion

The findings of this study indicate that the CLeD-EX is an effective and feasible instrument to promote key collaborative learning behaviours in medical students. Students indicated that the CLeD-EX was beneficial in terms of improving their collaborative learning and that the feedback provided through this process was useful. The qualitative findings illustrate how the CLeD-EX has a positive impact on learning.

Learners often report receiving insufficient feedback from tutors and whatever feedback is received is often not helpful, timely or constructive [41]. Important reasons for these findings could be time constraints faced by teachers or lack of experience or structure in providing feedback to their students. CLeD-EX in small group learning could provide the framework for tutors to engage with the students who need feedback the most; that is the 2–15 min time range implies that two minutes can be sufficient in many cases, but tutors can choose to spend additional time with one or two students who need the extra attention. Therefore, CLeD-EX aimed to address these issues by developing a process that would minimise the extra time load imposed on tutors, while providing a framework to give students feedback on these aspects. The feedback facilitated by the CLeD-EX included both written comments and oral dialogue with the tutor. In this study, only the written comments were analysed. Students may have received constructive comments on other aspects of their collaborative behaviours during the feedback dialogue.

The internal consistency of the CLeD-EX was greater in the tutor evaluation scale. The low Cronbach alpha in the student evaluation scale might be related in part to low sample size in the field-test. More likely however, the differences are more likely to be related to subjective judgements made by students in the student evaluation scale, whereas in the tutor evaluation scale, tutors utilise observable evidence to determine whether students are demonstrating collaborative skills, e.g. listening.

There was a statistically significant difference between the mean summed score of students’ self-evaluation and mean summed score of tutors’ evaluations of collaborative behaviours, with a large effect size. Direct comparison of mean self-evaluation and mean tutor-evaluation revealed a consistent under-evaluation by students. The literature also reports poor correlation between self-evaluation and tutor evaluation in a small group learning context [17, 42]. Moreover, there was a low correlation between the sum scores of individual items on self-evaluation and sum scores of individual items on tutor evaluation. Although there is a difference between student self-evaluation (more stringent) and tutor evaluation (more lenient), the items may be ranked similarly. There is a tendency for weaker students to overestimate their work and for better students to underestimate their performance [43–45]. Therefore, students’ self-evaluation may vary in accuracy. Self-assessment may thus be of limited value, if our aim is accuracy. However, in the context of developing essential professional skills of self-evaluation, self-assessment could play a significant role. Thus, self-assessment could be valuable in itself, but could play an enhanced role when paired with a peer or tutor assessment.

Comparisons between students’ pre-test and post-test indicated a significant improvement in students’ self-directed learning, critical thinking and group process interactions in the intervention group. In contrast, only the self-direction subscale improved in the control group, with a small effect size. The significant improvement of students on critical thinking and group process subscales in the intervention group provides evidence of the efficacy of the CLeD-EX.

Learning-oriented interactions in small group learning environments are reported to be constructive, collaborative and self-regulatory [46]. In the evaluation of CLeD-EX as an educational instrument, we explored the impact of the instrument on students’ self-directed learning, their ability to think critically and to interact within the group. Self-directed learning is defined as “a form of education that involves the individual learner’s initiative to identify and act on his or her learning needs (with or without assistance), taking increased responsibility for his or her own learning” [16]. The metacognitive skills for self-directed learning includes planning, monitoring progress and evaluating whether goals are achieved [47]. Enhancements in self-directed learning skills are known to positively influence deeper approaches to learning [48]. Furthermore, the focused design of the CLeD-EX with feedback and reflection, promoted students’ self–awareness of the key aspects which need to be considered during collaborative learning.

Another important finding in the evaluation of the CLeD-EX was the significant improvement in students’ self-reported critical thinking. The ability to think critically is vital to medical practice and its active training has to be part of the medical curriculum [49]. The CLeD-EX components of self-evaluation, tutor evaluation and feedback, followed by reflection might have triggered a cycle of critical thinking in students to develop their collaborative learning skills. Those processes are also likely to have led to the significant improvement in group process reported in the intervention group.

Lastly, the importance of reflection in medical education and building this reflective capacity in learners is well-established. Focused and guided approaches to develop reflective capability is encouraged at all stages of medical education [50]. Also, the qualitative findings illustrate how the CLeD-EX supported students to develop their skills in critical reflection and develop plans for further improvement.

### Limitations

The voluntary nature of the field-test of the CLeD-EX, with no protected time allocated to utilise the CLeD-EX, limited the number of student participants. The challenges of recruiting participants for research [51], especially educational research [52] are well–recognised. In addition, administering the CLeD-EX required tutor evaluation and feedback, requiring time and effort by tutors. All these aspects need to be considered when designing future strategies to implement CLeD-EX.

### Practical implications of CLeD-EX

The CLeD-EX can be used within formative evaluation activities in small group, collaborative learning contexts. This instrument focuses on the key collaborative learning behaviours and is designed of individual learner development within learning groups. In the field-test, the CLeD-EX was found to be especially relevant for junior medical students. It is suitable to be administered during the early stages of a Medicine program, when students are beginning to participate in small group learning activities. It has the potential to be used iteratively to develop students’ collaborative learning behaviours. Moreover, the structured design of the CLeD-EX can aid students by providing an opportunity to self-evaluate, to solicit and accept feedback and to self-reflect.

It is important to note that CLeD-EX needs to be implemented within a safe learning environment, where students will value constructive feedback. This will encourage students to take a genuine approach to evaluation, seeking and giving feedback to each other, and engaging in reflection.

## Conclusion

In conclusion, the CLeD-EX is a feasible, acceptable and effective educational instrument which focuses on developing key behaviours required for collaborative learning in medical students. Preliminary evaluation of the CLeD-EX indicates it has a positive impact on students’ critical thinking and collaborative learning behaviours.

### Future directions for CLeD-EX

Further research is needed into the acceptability of the CLeD-EX, and students and tutors’ experience of using the CLeD-EX. In addition, it will be worthwhile to explore its potential application within other healthcare disciplines such as dentistry, nursing, and allied health. The CLeD-EX could also be extended to postgraduate healthcare education, in which small group, collaborative learning is employed.

## Supplementary information

**Additional file 1.** CLeD-EX Instrument.

## Data Availability

The data that support the findings of this study are available from University of New South wales but restrictions apply to the availability of these data, which were used under license for the current study, and so are not publicly available. Data are however available from the authors upon reasonable request and with permission of University of New South Wales.

## Abbreviations

CLeD-EX: Collaborative Learning Development Exercise
CT: Critical thinking
GP: Group processing
SDL: Self-directed learning
