# Supplementary material for: The collaborative learning development exercise (CLeD-EX): an educational instrument to promote key collaborative learning behaviours in medical students
Source: BMC Med Educ. 2020 Mar 2;20:62. doi: 10.1186/s12909-020-1977-0 (PMC7052979; doi:10.1186/s12909-020-1977-0)
Supplement: Supplementary file 1 — Additional file 1. CLeD-EX Instrument. [file 12909_2020_1977_MOESM1_ESM.pdf]

# Collaborative Learning Development Exercise Tool (CLeD-EX)

## Part 1: Self-Evaluation

Approval No. 2014-7-39

| **Student Code** | C- |
|---|---|

| **Assessor** | Self |
|---|---|

| **Learning setting** | Scenario group learning- Phase 1 UNSW Medicine |
|---|---|

| **Collaborative behaviour during scenario group learning** | **Never** | **Rarely** | **Often** | **Always** | **Unable to assess. Please comment** |
|---|---|---|---|---|---|
| I am well-prepared for the learning sessions | ○ | ○ | ○ | ○ | |
| I am willing to work and I am engaged in learning activities | ○ | ○ | ○ | ○ | |
| I am involved in discussion and debate on different ideas | ○ | ○ | ○ | ○ | |
| I listen to others' points of view | ○ | ○ | ○ | ○ | |
| I share information with group members and voice my opinions | ○ | ○ | ○ | ○ | |
| I reflect on the feedback I receive and respond appropriately | ○ | ○ | ○ | ○ | |
| I find that engaging in collaborative learning enhances the quality of my learning | ○ | ○ | ○ | ○ | |

Overall, my skills in collaborative learning are well developed: YES ○ NO ○

**Email Facilitator**

## Part 2: Tutor Evaluation

| **Assessor** | Tutor |
|---|---|

| **Learning setting** | Scenario group facilitation- Phase 1 UNSW Medicine |
|---|---|

| **Collaborative behaviour during scenario group learning** | **Never** | **Rarely** | **Often** | **Always** | **Unable to assess. Please comment** |
|---|---|---|---|---|---|
| This student is well-prepared for learning sessions | ○ | ○ | ○ | ○ | |
| This student is willing to work and is engaged in learning activities | ○ | ○ | ○ | ○ | |
| This student is involved in discussion and debate on different ideas | ○ | ○ | ○ | ○ | |
| This student listens to others' points of view | ○ | ○ | ○ | ○ | |
| This student shares information with group members and voices his/her opinions | ○ | ○ | ○ | ○ | |
| This student reflects on the feedback they receive and responds appropriately | ○ | ○ | ○ | ○ | |
| This student demonstrates behaviours which enhance collaborative learning within the group | ○ | ○ | ○ | ○ | |

Overall, this student demonstrates skills which enhance the quality of collaborative learning: YES ○ NO ○

**Feedback to the student:**

| **Positive aspects of collaboration** | **Areas that can be improved** |
|---|---|
| | |

Time taken for feedback: minutes

**Assessor signature**: **Date:**

If you are willing to be interviewed about your experience in administering the CLeD-EX, please provide your email below. We will then send you the relevant Participant Information Statement and Consent Form and arrange convenient time for an interview. **Assessor Email**:

**Email Student**

## Part 3: Student Reflection

**(To be completed by the student after discussion with the assessor)**

| |
|---|
| Issues which were raised in this exercise that need to be addressed (to improve my collaborative learning behaviours): |
| Action plan for improvement: |

I found the CLeD-EX helped improve my skills in collaborative learning: YES ○ NO ○

I was provided with useful feedback through the CLeD-EX process: YES ○ NO ○

**FINAL SUBMISSION**

## Instructions for Student:

- In part 1 of the CLeD-EX form, please self-evaluate your skills in collaborative learning during the scenario group sessions, using the rating scale provided. This is a formative exercise, please be realistic in your rating.
- After completing your section, please arrange a 5-10 minute meeting with your facilitator, where they will evaluate your skills. You facilitator may choose to complete their evaluation and feedback in the same session or they may want to keep the form, complete their evaluation for you and provide you with feedback at a later time. Please discuss with your facilitator about their preference.
- Please listen to the feedback that is provided and take notes. You may discuss with your facilitator and develop an action plan to further improve your collaborative skills.
- In Part 3 please reflect on the feedback that is provided; you can discuss the issues that were raised and your personal action plan to improve your collaborative learning. Please also answer the questions about your experience using the CLeD-EX tool.
- Please submit the completed form online or via the email to Maha Iqbal (email: m.pervaziqbal@unsw.edu.au )
- Please retain a copy of the completed CLeD-EX form. You can include this completed form as "evidence of achievement" for the Teamwork capability in the phase 1 portfolio.

## Instructions for assessors:

- The CLeD-EX is a formative assessment instrument focussing on the collaborative behaviours which promote learning in small groups.
- The CLeD-EX will be completed after a period of observing the medical student's performance during the scenario group sessions.
- Please rate the student's performance on each behaviour using the scale provided.
- After the rating, please provide feedback to the student. This feedback may help the student to identify their strengths in collaborative learning, areas that can be improved and develop an action plan to further improve skills in collaborative learning. You have the option of rating the student and providing feedback in a single session or you may choose to complete the student rating and later discuss the feedback with the student.
- Please sign the completed form and return to the student. The student will then reflect on the rating and feedback received.
